# Fracture-induced amorphization of polycrystalline SiO_2_ stishovite: a potential platform for toughening in ceramics

**DOI:** 10.1038/srep06558

**Published:** 2014-10-09

**Authors:** Norimasa Nishiyama, Fumihiro Wakai, Hiroaki Ohfuji, Yusuke Tamenori, Hidenobu Murata, Takashi Taniguchi, Masafumi Matsushita, Manabu Takahashi, Eleonora Kulik, Kimiko Yoshida, Kouhei Wada, Jozef Bednarcik, Tetsuo Irifune

**Affiliations:** 1 Deutsches Elektronen-Synchrotron (DESY), Notkestr. 85, 22607 Hamburg, Germany; 2 Precursory Research for Embryonic Science and Technology (PRESTO), Japan Science and Technology Agency (JST), Chiyoda-ku, Tokyo 102-0075, Japan; 3 Secure Materials Center, Materials and Structures Laboratory, Tokyo Institute of Technology, R3-23 4259 Nagatsuta, Midori-ku, Yokohama 226-8503, Japan; 4 Geodynamics Research Center, Ehime University, 2-5 Bunkyo-cho, Matsuyama 790-8577, Japan; 5 Japan Synchrotron Radiation Research Institute/SPring-8, 1-1-1 Kouto, Sayo, Hyogo 679-5198, Japan; 6 National Institute for Materials Science, 1-1 Namiki, Tsukuba 305-0044, Japan; 7 Department of Mechanical Engineering, Ehime University, 2-5 Bunkyo-cho, Matsuyama 790-8577, Japan; 8 National Research Nuclear University (MEPhI), Kashirskoe shosse 31, Moscow, 115409, Russia; 9 Fuji Die Co., Ltd., 2-17-10 Shimomaruko, Ohta-ku, Tokyo 146-0092, Japan; 10 Earth-Life Science Institute, Tokyo Institute of Technology, 2-12-1-1E-1 Ookayama, Meguroku, Tokyo 152-8500, Japan

## Abstract

Silicon dioxide has eight stable crystalline phases at conditions of the Earth's rocky parts. Many metastable phases including amorphous phases have been known, which indicates the presence of large kinetic barriers. As a consequence, some crystalline silica phases transform to amorphous phases by bypassing the liquid via two different pathways. Here we show a new pathway, a fracture-induced amorphization of stishovite that is a high-pressure polymorph. The amorphization accompanies a huge volume expansion of ~100% and occurs in a thin layer whose thickness from the fracture surface is several tens of nanometers. Amorphous silica materials that look like strings or worms were observed on the fracture surfaces. The amount of amorphous silica near the fracture surfaces is positively correlated with indentation fracture toughness. This result indicates that the fracture-induced amorphization causes toughening of stishovite polycrystals. The fracture-induced solid-state amorphization may provide a potential platform for toughening in ceramics.

Silicon dioxide (silica) has many polymorphs. The stable phase at ambient conditions is α-quartz, and this phase transforms into three different phases with temperature before it melts. A number of pressure-induced transformations are known. α-quartz transforms to coesite at ~2 GPa. In all the crystalline phases stable at pressures below coesite (including coesite), silicon atoms are tetrahedrally coordinated by oxygen atoms. Silica glass, which is a metastable phase, also has a silicon coordination number (CN) of four. Stishovite^1^ is stable at pressures above ~9 GPa (ref. 2) with a CN = 6. Stishovite undergoes further transformations to three different stable phases (CN = 6) with pressure^3^,^4^,^5^ up to 300 GPa.

The complexity of phase relations in silica arises from the presence of many metastable phases^6^. The appearance of the metastable phases depends strongly on the initial structure and pressurization-annealing procotol^6^. α-quartz has been reported to transform to several intermediate metastable crystalline phases^7^,^8^,^9^ via room-temperature compression before it amorphizes^10^. Cristobalite, which is a high-temperature phase, was reported to transform to a stable phase via a metastable crystalline phase (hp-cristobalite^11^) by means of room-temperature compression^12^,^13^. It was also reported that amorphous silica crystalizes via a metastable crystalline phase^14^ under pressure. The presence of many metastable crystalline phases implies that large kinetic barriers exist between the stable phases^6^.

Solid-state amorphization, in which a crystal is transformed to an amorphous phase by bypassing the liquid, is also a consequence of large kinetic barriers. In silica, two pathways are known. The first is pressurization^10^: α-quartz and coesite are amorphized by compression at 25–35 GPa and room temperature. The second is heating of high-pressure phases at 1 bar: coesite and stishovite transform to an amorphous phase at ~1100 and ~550°C, respectively^15^, prior to stable phases (quartz and cristobalite).

In the present study, we show evidences of a new pathway, a fracture-induced solid-state amorphization of polycrystalline stishovite, by careful observations of fracture surfaces of samples with different grain sizes using electron microscopy and X-ray absorption near edge structure (XANES) spectroscopy. An unexpected consequence arises from the fracture-induced amorphization of stishovite: it may trigger toughening of this ceramic.

## Results

### Synthesis of stishovite polycrystals

A series of stishovite polycrystals (SPs) were synthesized at every 100°C between 1200 and 2000°C and at a fixed pressure of 15 GPa. We used a rod-shaped bulk silica glass with no visible void as a starting material. The chemical composition is pure: all the impurities are less than 0.1 ppm except OH (~800 ppm). X-ray diffraction measurements of the recovered samples were performed using synchrotron radiation. Crystallite size was refined by full profile analysis by the Rietveld method. The obtained crystallite sizes were consistent with the mean grain sizes measured by electron and optical microscopy. The crystallite size (CS) increases monotonically with temperature from ~100 nm at a synthesis temperature (*T*_S_) of 1200°C to ~30 μm at *T*_S_ = 2000°C.

### Transgranular and intergranular fractures

**Figure 1:**
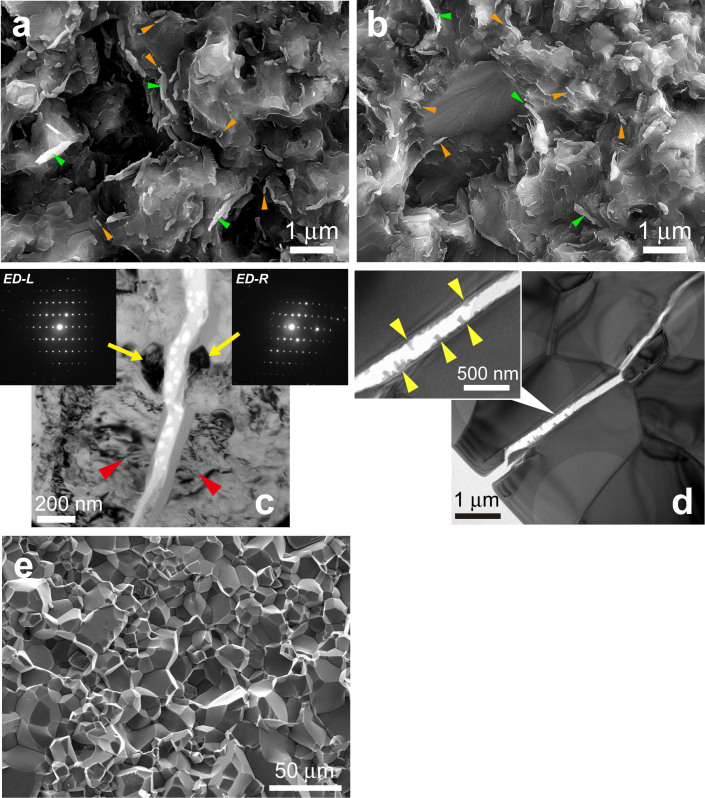
Microstructures of fracture surfaces. (a), (b), Secondary electron images (SEIs) of a fracture surface of a sample synthesized at 1300 (a) and 1600°C (b). Orange arrows, small “worms”; green arrow, long “worms” with length > 1 μm. (c), (d), TEM micrographs of cracks produced by Vickers indentations for samples synthesized at 1200 (c) and 1600°C (d). c, Two electron diffraction patterns of grains on the left-hand side (ED-L) and on the right-hand side (ED-R) of the crack are shown. These two grains have the very similar crystallographic orientations, indicating that transgranular fracture occurs in this sample with crystallite size of ~250 nm. Other two grains (red arrows) divided by the crack seem also to be a single grain before the cracking. (d), Transgranular fractures are observed. The inset shows a magnified view of the crack indicated by the white arrow. The bosses indicated by yellow arrows can be worm-like textures (b). (e), A SEI of a fracture surface of a sample synthesized at 2000°C. Intergranular fracture is dominant.

We observed fracture surfaces with a field emission scanning electron microscope (FESEM). A fracture surface of a SP (*T*_S_ = 1300°C, CS ~ 250 nm) shows a rough and distinctive morphology (Fig. 1a). The presence of many worm-like^16^ textures was observed and the whole fracture surface is completely filled with this texture (supplementary information). The dimensions of a “worm” are several tens and hundreds of nanometers in diameter and length, respectively (orange arrows). The presence of long “worms” (>1 μm long) can also be seen (green arrows). We observed similar features on a fracture surface of a SP with *T*_S_ = 1600°C and CS ~ 1.5 μm (Fig. 1b). All the SPs with *T*_S_ below 1600°C show similar fracture surfaces.

In order to observe crack propagation in these SPs, we made thin sections whose surfaces are perpendicular to cracks produced by Vickers indentations and the thin sections were observed by a transmission electron microscope (TEM). We observed grains showing transgranular fractures in SPs synthesized at 1200°C (Fig. 1c) and 1600°C (Fig. 1d). Grains showing transgranular and intergranular fractures may be present on the fracture surfaces of these SPs, but it is difficult to evaluate the ratio of grains showing these two fractures by observing the fracture surfaces (Fig. 1a, b). Since we observed extensive transgranular fractures in these SPs, the formation of worm-like textures might be related to the transgranular fractures. These results show that worm-like textures exist and transgranular fracture occurs in SPs with *T*_S_ below 1600°C.

On the other hand, a fracture surface of a SP with *T*_S_ = 2000°C resembles that of alumina ceramics^17^ (Fig. 1e). This image clearly shows that intergranular fracture is dominant. Our results show that the ratio of stishovite grains showing transgranular and intergranular fractures changes with *T*_S_: transgranular fractures occur extensively at *T*_S_ below 1600°C; intergranular fracture is dominant at *T*_S_ = 2000°C.

### XANES measurements: CN change induced by transgranular fracture

**Figure 2:**
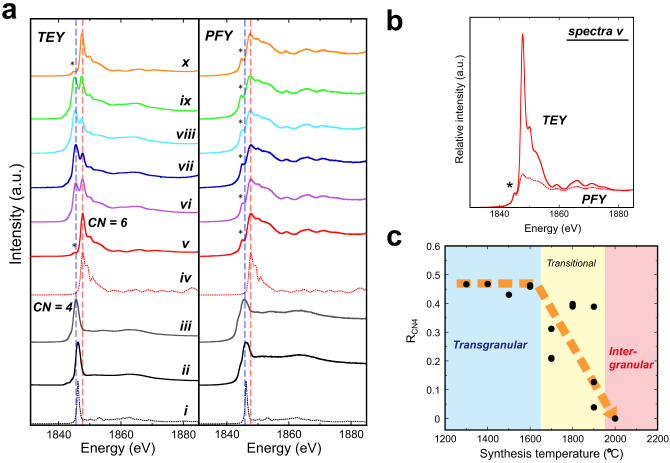
Results of XANES measurements. (a), Representative XANES spectra and those obtained by first-principles calculations. TEY and PFY spectra collected simultaneously from one measurement are shown side by side. (i), α-quartz by first-principles calculation; (ii), ordinary silica glass; (iii) densified silica glass; (iv), stishovite by first-principles calculation; (v), unpolished surface of a polycrystalline stishovite sample with synthesis temperature (*T*_S_) of 1300°C; (vi), polished surface *T*_S_ = 1300°C; (vii), fracture surface *T*_S_ = 1300°C; (viii), fracture surface *T*_S_ = 1600°C; (ix), fracture surface *T*_S_ = 1800°C; (x) fracture surface *T*_S_ = 2000°C. *, a pre-edge peak of stishovite. All the spectra are normalized by using the peak-top intensity. The blue and red dashed-lines represent peak positions of densified silica glass (CN = 4, iii) and of stishovite (CN = 6, v), respectively. (b), Relative intensities of TEY (the solid line) and PFY (the dot-line) spectra of an unpolished surface of a polycrystalline stishovite (*T*_S_ = 1300°C, spectra v). *, a pre-edge peak of stishovite. (c), A relation between ratio of the CN = 4 materials on the fracture surfaces of polycrystalline stishovite (R_CN4_) and synthesis temperature. The dashed orange arrow is a visual guide. “Transgranular”, transgranular fractures are extensively observed; “Intergranular”, intergranular fracture is dominant.

In order to examine phenomena that happened on fracture surfaces of SPs, we performed Si-K XANES measurements (Fig. 2a). For each measurement, we collected two spectra simultaneously: a spectrum collected by the total electron yield (TEY) method and that by the partial fluorescence yield (PFY) method. The former is a surface-sensitive method: a XANES spectrum to a depth of ~10 nm from the surface^18^ can be obtained. The latter is a bulk-sensitive method: a spectrum to a depth of ~1 μm can be obtained for silica materials. Since the peak position of XANES spectra of stishovite with CN = 6 is quite different from those of silica materials with CN = 4, these spectra can be used as fingerprints^19^,^20^ to distinguish silica materials with different CNs.

First, we performed a measurement for an unpolished (as sintered) surface of a SP (*T*_S_ = 1300°C, CS ~ 250 nm). The TEY and PFY spectra (v) can be explained by the presence of a single phase of stishovite. Relative intensities of the TEY and PEY spectra (v) are shown in Fig. 2b. The peak intensities of the PFY spectrum are lower than those of the TEY spectrum because of the self-absorption effect. A pre-edge peak that may be attributed to the effect of the atomic vibrations at room temperature^21^ is observed in both spectra (Fig. 2b).

On the other hand, two peaks indicating the presence of materials with CN = 4 and 6 were clearly observed in a TEY spectrum obtained from a polished surface (by diamond pastes down to a grain size of 0.25 μm) of the SP (vi). Note that the CN = 4 peak cannot be observed in the PFY spectrum. These results demonstrate that the CN = 4 materials coexist with stishovite (CN = 6) in a thin layer whose thickness is several tens of nanometers from the polished surface. A previous study reported that stishovite was inverted to silica glass (CN = 4) by mechanical grinding^22^, which supports our observations. Similar features were observed in the spectra obtained from fracture surfaces of SPs (*T*_S_ = 1300 and 1600°C) in which transgranular fractures occur and worm-like textures exist (vii, viii), demonstrating the coexistence of the CN = 4 material with stishovite (CN = 6) in thin layers (several tens of nanometer thick) adjacent to the fracture surfaces.

The presence of the CN = 4 phase near the fracture surfaces can only be detected by using surface-sensitive methods. In a previous study^16^, the presence of the thin CN = 4 layer was undetectable using conventional Raman spectroscopy (supplementary information) because penetration depth of visible light into SPs is much deeper (they look translucent) than the thickness of the CN = 4 layer (several tens of nanometers). In the present study, the TEY-XANES method enabled us to detect this thin layer.

We observed a decrease of relative intensity of the CN = 4 peak with respect to the CN = 6 peak intensity in TEY spectra with increasing synthesis temperature (*T*_S_) above 1700°C (ix). At the highest *T*_S_ of 2000°C, we observed the complete absence of the CN = 4 peak (x). As shown in Fig. 1e, intergranular fracture is dominant in this SP.

We estimated ratios of the CN = 4 materials (R_CN4_) in the thin layers adjacent to the fracture surfaces by comparing the TEY spectra with synthetic ones by first-principles calculations and obtained a plot showing R_CN4_ vs. *T*_S_ (Fig. 2c). R_CN4_ is almost constant in a temperature range where transgranular fractures and worm-like textures are observed (*T*_S_ below 1600°C), whereas R_CN4_ decreases with temperature at *T*_S_ > 1700°C and it reaches zero (absence of the CN = 4 phase) at *T*_S_ = 2000°C where intergranular fracture is dominant.

### Fracture-induced solid-state amorphization

**Figure 3:**
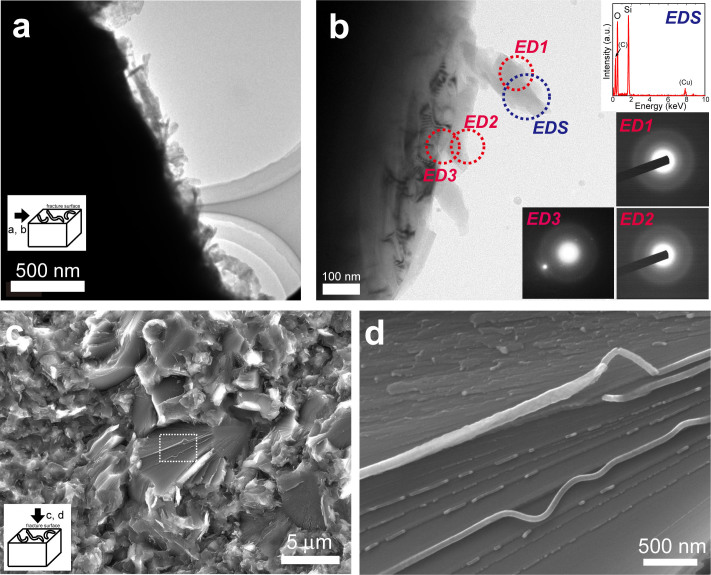
Microstructures of amorphous silica produced by fracture of a stishovite polycrystal synthesized at 1600°C. (a), A low-magnification TEM micrograph of a fragment of a mechanically crushed sample. The fracture surface is perpendicular to the image. Worm-like textures are observed. (b), A magnified TEM image of worm-like textures. Electron diffraction patterns (ED1 and 2) and an X-ray fluorescence spectrum by energy dispersive X-ray spectroscopy (EDS) show presence of an amorphous silica phase on the fracture surface. Carbon (C) and copper (Cu) peaks are from a TEM grid. An electron diffraction pattern (ED3) that is collected at the interface between the amorphous phase and stishovite shows coexistence of halo and diffracted spots. (c), A SEI of a fracture surface. Presence of large fractured grains (diameter > 5 μm; e.g. the grain at the center of this image) was observed. These grains allow us to observe an intergranular fracture surface of a single stishovite grain. (d), A magnified SEI of the area indicated by the dashed square in c. Amorphous silica materials are localized on fractured stishovite grains.

In order to identify the CN = 4 phase, we performed TEM observations. A TEM sample was carefully prepared from a SP (*T*_S_ = 1600°C, CS ~ 1.5 μm) solely by mechanical crushing. A low magnification image in which a crushed surface is perpendicular to the image shows the presence of worm-like textures (Fig. 3a). Selected-area electron diffraction (SAED) patterns of the worm-like textures have a halo and an example of X-ray fluorescence spectra show the presence of only silicon and oxygen (Fig. 3b). These results indicate that fracture of this SP induces transformation from stishovite (CN = 6) to amorphous silica (CN = 4), which results in the coexistence of the CN = 4 and CN = 6 peaks in the TEY-XANES spectrum (viii in Fig. 2a). These observations were carefully conducted using a very gentle electron beam (irradiation current: 50–100 pA) to avoid any potential beam damage^23^. The SAED patterns were taken with long exposure time (20 s). Elemental analysis was also performed using a defocused gentle beam (~50 pA). There are two possible materials as the fracture-induced amorphous silica: ordinary silica glass (o-glass) and densified silica glass^24^ (d-glass). A previous study^25^ reported that stishovite transforms to an amorphous phase that exhibits features of the d-glass by heating at 1 bar. Although our TEY-XANES spectra obtained from fracture surfaces of SPs can be explained better by using the spectrum of a d-glass (Fig. 2a), further studies will be needed to show that conclusively.

In order to locate the amorphous silica materials on a fracture surface of this sample (*T*_S_ = 1600°C, CS ~ 1.5 μm), we performed FESEM observations. Many string-shaped materials were observed (Fig. 3d) on fractured stishovite grains (Fig. 3c). These strings are often curved and bended (Fig. 3d), suggesting that these are amorphous silica deformed by a huge volume expansion (~95% from stishovite to o-glass; ~60% from stishovite to d-glass) and by heat produced by exothermic transition from stishovite to an amorphous phase^26^. The curved strings that are expected to be amorphous silica are localized on fractured stishovite grains (Fig. 3d). These strings could be one of the sources of the worm-like texture (Figs. 1a, 1b, and 3a). Note that the worm-like texture cannot be the only form of amorphous silica produced by fracture. Since the worm-like textures are scattered on the fracture surfaces (Figs. 1a and 1b) and the amount of this texture does not seem to be enough to explain R_CN4_ of ~ 0.45 (Fig. 2c), the amorphized silica materials may exist in stishovite grains and/or grain boundaries near the fracture surfaces. The results by TEM and FESEM observations (Figs. 3b and 3d) are consistent with those by the TEY-XANES spectra. Our experimental results show that fracture of SPs induces solid-state amorphization of stishovite (amorphization was not detected in the SP with *T*_S_ = 2000°C) and that the formed amorphous silica (CN = 4) coexists with stishovite (CN = 6) in thin layers whose thickness is several tens of nanometers from the fracture surfaces.

### Metastability of stishovite

**Figure 4:**
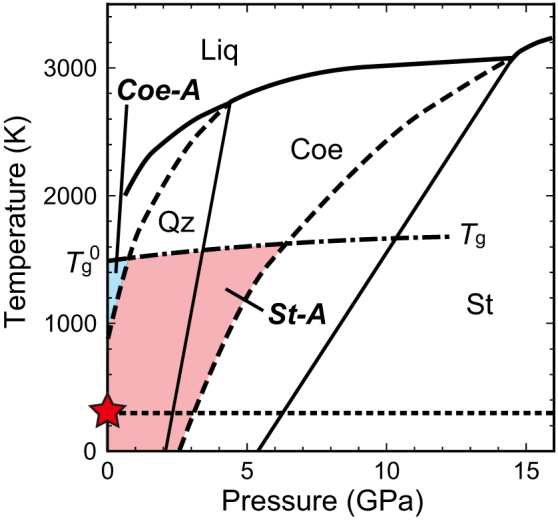
Schematic illustration to show metastability of stishovite. Bold lines represent equilibrium phase boundaries^2^. The dot-line represents room-temperature. The dot-dash line represents glass transition temperature (*T*_g_); *T*_g_^0^: *T*_g_ at 1 bar. Dashed lines represent hypothetical metastable extensions of melting curves of coesite and stishovite. The metastable melting curve of coesite is a smooth interpolation between a triple point (quartz, coesite, liquid) and *P*-*T* conditions at 1 bar and 875 K where Δ*G* between coesite and o-glass is zero^27^. The metastable melting curve of stishovite is a smooth interpolation between a triple point (coesite, stishovite, liquid) and *P*-*T* conditions at 3 GPa and room temperature (Δ*G* between stishovite and o-glass is zero). *P*-*T* conditions where solid-state amorphizations of coesite and stishovite are energetically favorable are shown as the blue area. *P*-*T* conditions where solid-state amorphization of stishovite is energetically favorable are shown as the pink area. The red star represents atmospheric pressure and room-temperature conditions. Qz, quartz; Coe, coesite; St, stishovite; Liq, liquid; Coe-A, coesite-amorphous transformation; St-A, stishovite-amorphous transformation.

Richet^27^ interpreted solid-state amorphization from a thermodynamical point of view: an amorphous phase is energetically more stable than a crystalline phase at temperatures between the glass transformation temperature (*T*_g_) (the upper bound) and the temperature (*T*_f_) at which the Gibbs free energy difference between crystal and glass (Δ*G*) is zero (the lower bound). Richet^27^ pointed out that the amorphous state is energetically more favorable than stishovite at ambient conditions because Δ*G* < 0 (stishovite is unstable with respect to o-glass) at 1 bar and 0 K. We calculated a pressure where Δ*G* = 0 between stishovite and o-glass at room temperature using previously determined thermodynamics parameters^28^,^29^. (We were unable to calculate Δ*G* = 0 between stishovite and d-glass because of the lack of enthalpy measurement of d-glass.) The calculated pressure was 3 GPa and pressure-temperature conditions where solid-state amorphization of stishovite is energetically favorable are shown in Fig. 4. At ambient conditions, stishovite is over-decompressed, but amorphization of stishovite is hindered by the large kinetic barrier. When stress to induce fracture is applied to stishovite, it can be amorphized by overcoming this kinetic barrier.

There have been many studies concerning the mechanisms of solid-state amorphization (e.g. ref. 30). However, our experimental results are not enough to demonstrate which mechanism controls the fracture-induced amorphization. Further experimental and theoretical studies are required to elucidate the mechanism of this amorphization process.

### Toughening by fracture-induced amorphization

The solid-state amorphization of stishovite is a fracture-induced transformation with volume expansion. The volume expansion from stishovite to amorphous silica is 60–95%. The best known material to exhibit fracture-induced transformation with volume expansion is partially-stabilized zirconia (PSZ). Fracture of PSZ induces a tetragonal-to-monoclinic (t-m) transformation^31^ with volume expansion of about 4%. Since the t-m transformation in PSZ induces toughening, we hypothesize that fracture-induced amorphization of stishovite causes toughening of SPs.

In order to verify this hypothesis, fracture toughness of SPs should be measured. Fracture toughness of ceramics can accurately be measured^32^,^33^ by a chevron-notched beam (CNB), a single-edge pre-cracked beam (SEPB), or a surface crack in flexure (SCF) method. For these tests, large specimens (e.g., 3 × 4 × 45 mm) are usually required. However, our synthesized samples (~2 mm in diameter and ~1.2 mm in thickness) are much smaller than the required sample size for accurate measurements. Thus, there is no alternative but to evaluate fracture toughness by the Vickers indentation fracture (IF) method^16^.

**Figure 5:**
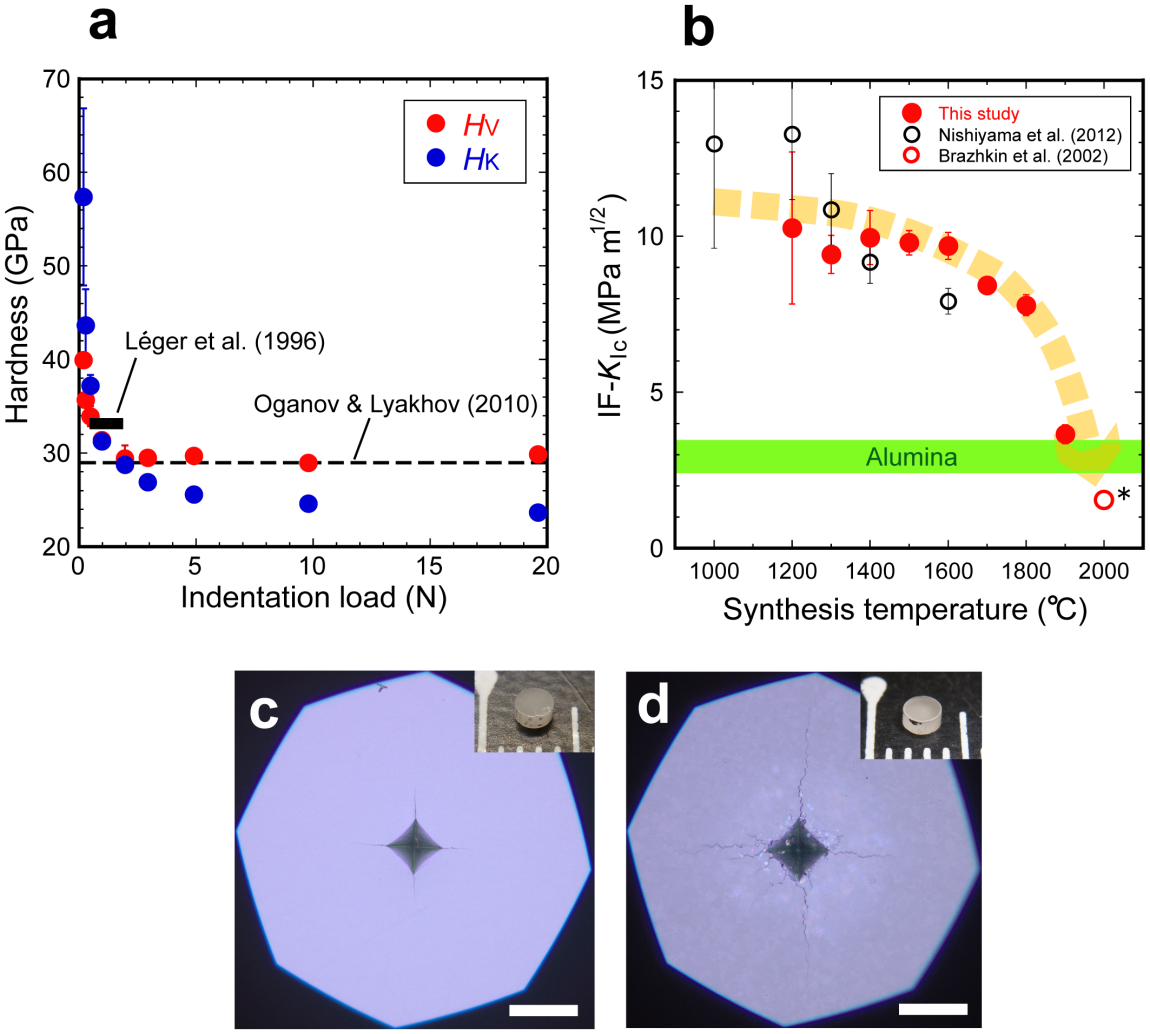
Hardness and toughness of stishovite polycrystals determined by indentation tests. (a), Hardness as a function of indentation load for a SP synthesized at 1300°C. *H*_V_, Vickers hardness; *H*_K_, Knoop hardness. At each load, the value was obtained by averaging three independent results. Léger and others^34^ determined *H*_K_ = 33 GPa by averaging the data obtained at indentation loads of 0.49, 0.98, and 1.96 N (the black solid bar). The dashed line, a result by theoretical calculation^36^. (b), Vickers indentation fracture toughness (IF-*K*_Ic_) as a function of synthesis temperature (*T*_S_). *, we assumed that IF-*K*_Ic_ of a sample with *T*_S_ = 2000°C is 1.6 MPa m^1/2^ that is equal to that of the single crystal^38^. Fracture toughness of polycrystalline alumina is also shown as the green area for comparison. The dashed orange arrow is a visual guide. (c), An optical micrograph of a Vickers indentation trace of a SP synthesized at 1300°C (crystallite size, ~250 nm): Vickers hardness (*H*_V_), ~29.5 GPa; IF-*K*_Ic_ (49-196 N), 9.4 ± 0.6 MPa m^1/2^. (d), An optical micrograph of a Vickers indentation trace of a SP synthesized at 1900°C (crystallite size, ~20 μm): *H*_V_, ~21.2 GPa; IF-*K*_Ic_ (98 N), 3.7 ± 0.3 MPa m^1/2^. The insets show the whole views of the samples with diameter of ~2 mm and height of ~1.2 mm (c, d). The indentation traces were obtained at an applied load of 98 N (10 kgf) and the scale bars represent 100 μm (c, d).

Figure 5 shows results of Vickers indentation tests. Stishovite is the hardest known oxide that can be utilized at the ambient conditions^34^,^35^. Hardness values of a SP with *T*_S_ = 1300°C are consistent with the previous results by experiments^16^,^34^ and a theoretical calculation^36^ (Fig. 5a). Figure 5b shows IF-fracture toughness (*K*_Ic_) as a function of synthesis temperature. The present data below *T*_S_ of 1400°C are consistent with those of a previous study^16^ within experimental errors. The IF-*K*_Ic_ value of a SP with *T*_S_ = 1600°C is higher than that of the previous study. The IF-*K*_Ic_ values at *T*_S_ up to 1800°C are two to three times higher than those of alumina polycrystals^37^,^38^. IF-*K*_Ic_ of SPs rapidly decreases with *T*_S_ above 1900°C. We obtained IF-*K*_Ic_ of 3.7 ± 0.3 MPa m^1/2^ for a SP with *T*_S_ = 1900°C at an indentation load of 98 N, whereas a SP with *T*_S_ = 2000°C was broken into several pieces by an indentation at this load. These results indicate that the SP with *T*_S_ = 2000°C is more brittle than that with *T*_S_ = 1900°C. We therefore assumed that the IF-*K*_Ic_ of the SP with *T*_S_ = 2000°C is equal to that of single crystal^39^ (~1.6 MPa m^1/2^). These results suggest that stishovite is inherently brittle^39^, but SPs with *T*_S_ below 1600°C are highly toughened (>10 MPa m^1/2^). Of course, these are not accurate values, but micrographs of indentation traces of SPs synthesized at 1300°C (Fig. 5c) and 1900°C (Fig. 5d) also indicate that the former is tougher than the latter. It should also be noted that SPs with *T*_S_ below 1600°C can be simultaneously hard and tough (Vickers hardness: ~30 GPa; IF-*K*_Ic_: ~10 MPa m^1/2^). A new technique to measure *K*_Ic_ of these small samples accurately is highly required^32^ to show the simultaneous high-hardness and toughness conclusively.

**Figure 6:**
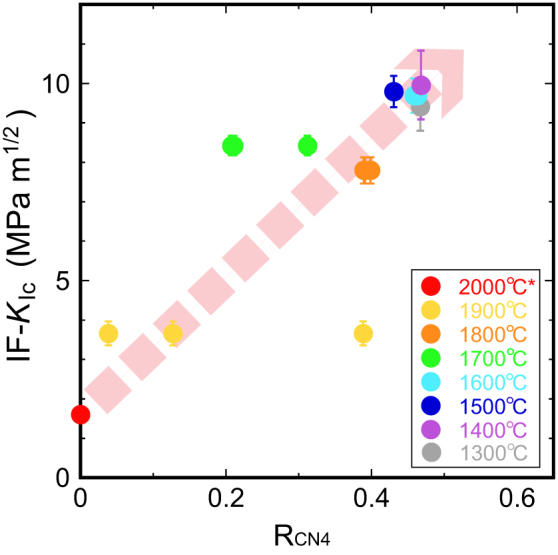
Experimental data to indicate that fracture-induced amorphization of polycrystalline stishovite triggers toughening. We plotted Vickers indentation fracture toughness (IF-*K*_Ic_) against the ratio of solid-state amorphized silica in a thin-layer (several tens of nanometers thick) adjacent to fracture surfaces of SPs estimated by TEY-XANES measurements (R_CN4_). *, we assumed that IF-*K*_Ic_ of a sample with *T*_S_ = 2000°C is 1.6 MPa m^1/2^ that is equal to that of the single crystal^39^. The dashed pink arrow is a visual guide.

In order to show the relationship between fracture-induced amorphization of stishovite and toughening of SPs, we ploted IF-*K*_Ic_ against the ratio of solid-state amorphized silica on fracture surfaces of SPs (R_CN4_) estimated by TEY-XANES measurements in Figure 6. IF-*K*_Ic_ increases with R_CN4_, which supports our hypothesis: fracture-induced amorphization of stishovite causes toughening of SPs.

A probable mechanism for the main contribution to the increase of IF-*K*_Ic_ of SPs is transformation toughening^40^. It was proposed^41^ that the increase of *K*_Ic_ (Δ*K*_Ic_) by the transformation toughening is expressed using the following equation: Δ*K*_Ic_ = 0.2143 *E* *V*_f_ *e*^T^ *h*^1/2^/(1-*ν*), where *E*, *V*_f_, *e*^T^, *h*, and *ν* are Young's modulus, volume fraction of the transformed material, volume expansion associated with the transformation, thickness of the transformed area, and Poisson's ratio, respectively. We calculated *h* using this equation with previously reported elastic moduli^16^, *V*_f_ = 0.45 (Fig. 6), and *e*^T^ = 0.6 (stishovite to d-glass) – 0.95 (stishovite to o-glass) to explain Δ*K*_Ic_ (~8.4 MPa m^1/2^), i.e. the increase from the IF-*K*_Ic_ of the single crystal (~1.6 MPa m^1/2^) to those of SPs with *T*_S_ below 1600°C (~10 MPa m^1/2^). The calculated *h* is 20–50 nm. This is consistent with our results obtained by XANES measurements (Fig. 2a). The thickness of the transformation zone of SPs could be at least one order of magnitude thinner than that of PSZ (*h* ~ 600 nm for an MgO-containing PSZ^40^). The transformation zone of PSZ can often be observed visually as an uplift surrounding an indentation trace^42^, whereas that of SPs is difficult to detect^16^ because it is only a nanolayer. Measurements of *K*_Ic_ as a function of crack extension are essential to understanding the mechanisms that contribute to the toughening of SPs. It is therefore highly required to develop a new technique to examine crack growth resistance behavior of these small samples, which will also allow us to measure *K*_Ic_ accurately.

## Discussion

Fracture-induced solid-state amorphization may provide a new platform to produce toughened ceramics. As discussed by Richet^27^, there are some other high-pressure minerals that exhibit crystal vitrification at low pressures (i.e. ~1 bar). Jadeite is an example. It is noteworthy that natural jadeite polycrystals, which are called as jade, have been well-known to be tough rocks (*K*_Ic_ ~ 7 MPa m^1/2^) with extensive transgranular cleavage^43^, which is similar to our SPs. Fracture-induced amorphization might be observed on the transgranular fracture surfaces of jade. Richet^27^ also discussed that high-pressure silicates with CN = 6 are prime candidates for low-pressure vitrification. The present results indicate that these materials can potentially be toughened by fracture-induced amorphization. A survey of tough ceramics from silicates or abundant minerals on the Earth meets the requirement for managing the scarcity of chemical elements^44^.

## Methods

### Sample synthesis under high-pressure and temperature

Polycrystalline stishovite samples were synthesized using a Kawai-type high-pressure apparatus (LPR 1000-400/50, Max Voggenreiter GmbH) installed in Photon Science, DESY. An octahedral pressure medium (Cr-doped magnesia) with edge length of 14 mm was compressed by tungsten carbide anvils with truncated edge length of 7 mm. A cylindrical LaCrO_3_ furnace and disk-shaped ZrO_2_ thermal insulators were employed. Generated pressure was calibrated as a function of applied load using pressure-fixed points (ZnTe, 9.6 and 12.0 GPa; ZnS, 15.6 GPa). Temperature was calibrated as a function of input power in a separate run using a W_0.95_R_0.05_-W_0.74_Re_0.26_ thermocouple. The dimensions of the starting material are 2.5 mm in diameter and 1.4 mm in height. A sample container made of pure magnesia and platinum foils was employed. The assembled sample container in which a starting material was enclosed was dried for more than two hours in an oil-free vacuum oven (~8 hPa, ~150°C). After drying, the sample container was embedded into the furnace and the high-pressure cell was assembled. Load was applied first up to the target value under room temperature and electric power was increased at the constant load. Temperature was increased rapidly from ~450°C to the target value within 30 s. We kept the target temperature constant for 30 min. Temperature was decreased to ~450°C and decompression (3h) was started. The decompression under high-temperature was beneficial to sample recovering without cracking. Some polycrystalline stishovite samples were synthesized using a Kawai-type apparatus (Orange-3000) installed in Geodynamics Research Center, Ehime University. A densified silica glass was synthesized using a Belt-type apparatus installed in National Institute of Materials Science. The synthesis conditions are 7.7 GPa and 1200°C. The starting material was the same as that used for the synthesis of polycrystalline stishovite with different dimensions: an ordinary silica glass disk with 5.5 mm in diameter and 2.0 mm in height was used.

### X-ray diffraction measurements using synchrotron

The measurements were performed at P02.1 (High-resolution Powder X-ray Diffraction Beamline), PETRAIII, Germany. X-rays monochromatized by a (111) diamond and a (111) silicon crystal in Laue geometry with an energy of ~60 keV were used. The incident beam-size was ~300 × 300 μm^2^. The transmission geometry was employed for the measurements. A two-dimensional detector (XRD 1621, PerkinElmer) was employed. The sample-to-detector distance (~750 mm) and the detector orthogonality were calibrated using a diffraction pattern of cerium dioxide powder.

### SEM and TEM observations

A field emission scanning electron microscope (JSM-7000F, JEOL) operating at 15 kV was used for SEI observations. Osmium coating was performed for all the samples. TEM observations were performed using JEM-2010 (JEOL) operating at 200 kV. Thin foils of the samples for the TEM observations (Fig. 1c, d) were prepared by focused ion beam technique using JEM-9310FIB (JEOL) operating at 30 kV with Ga ion beam. We used opposed tungsten carbide anvils to crush a fragment of a SP to prepare a TEM sample for Figs. 3a and b.

### XANES measurements and first-principles calculations

All the measurements were performed at BL27SU, SPring-8, Japan. Photon beam from a figure-8 undulator was dispersed by a soft X-ray monochromator with varied-line-spacing plane gratings. The beam diameter was ~500 μm at the sample positions. The intensity of the incoming beam was monitored by measuring the drain current on the surface of a post-focusing mirror. Photon energy was scanned by controlling the undulator gap and positions of the monochromator and slits simultaneously. Data were collected at every 0.1 eV with exposure time of 2 s between 1830 and 1885 eV. Theoretical Si-K XANES spectra were calculated using the first-principles method with a core-hole effect included. Details of conditions were shown in supplementary information.

### Mechanical properties measurements

Hardness and fracture toughness measurements were performed using a Vickers indenter (HV-114, Mitsutoyo) and a microhardness indenter (HM-221, Mitsutoyo). A hard steel standard with 900 HV was used. The top and bottom surfaces of the samples were mirror-polished by using diamond pastes down to 0.25 μm. IF-*K*_Ic_ was calculated from the measured crack length, c, using an equation proposed by Anstis et al.^45^. The values of Vickers hardness determined for each samples in the present study and Young's modulus determined in a previous study^16^ were employed for the calculations.

## Author Contributions

N.N. and F.W. designed the research. N.N., T.T., E.K., K.Y. and T.I. synthesized samples by high-pressure experiments. H.O. performed TEM observations and N.N. and H.O. performed FESEM observations. N.N., H.O., M.M., M.T. and T.I. discussed and interpreted images obtained by FESEM observations. N.N., Y.T., H.M. and M.M. performed XANES measurements. H.M. performed first-principles calculations of XANES spectra. N.N. and K.W. measured mechanical properties. N.N., E.K. and J.B. performed XRD measurements and analyzed the data. N.N., F.W., T.T., H.O. and H.M. wrote the paper.

## Supplementary Material

Supplementary Information supplementary information
