# Supplementary material for: Fracture-induced amorphization of polycrystalline SiO2 stishovite: a potential platform for toughening in ceramics
Source: Sci Rep. 2014 Oct 9;4:6558. doi: 10.1038/srep06558 (PMC4190503; doi:10.1038/srep06558)
Supplement: Supplementary Information [file srep06558-s1.pdf]

Supplementary information

Fracture-induced amorphization of polycrystalline SiO_2 stishovite: a potential platform for toughening in ceramics

Norimasa Nishiyama[1,2*], Fumihiro Wakai[3], Hiroaki Ohfuji[4], Yusuke Tamenori[5], Hidenobu Murata[6], Takashi Taniguchi[6], Masafumi Matsushita[7], Manabu Takahashi[7], Eleonora Kulik[1,8], Kimoko Yoshida[3], Kouhei Wada[9], Jozef Bednarcik[1], Tetsuo Irifune[4, 10]

1: Deutsches Elektronen-Synchrotron (DESY), Notkestr. 85, 22607 Hamburg, Germany
2: Precursory Research for Embryonic Science and Technology (PRESTO), Japan Science and Technology Agency (JST), Chiyoda-ku, Tokyo 102-0075, Japan
3: Secure Materials Center, Materials and Structures Laboratory, Tokyo Institute of Technology, R3-23 4259 Nagatsuta, Midori-ku, Yokohama 226-8503, Japan
4: Geodynamics Research Center, Ehime University, 2-5 Bunkyo-cho, Matsuyama 790-8577, Japan
5: Japan Synchrotron Radiation Research Institute/SPring-8, 1-1-1 Kouto, Sayo, Hyogo 679-5198, Japan
6: National Institute for Materials Science, 1-1 Namiki, Tsukuba 305-0044, Japan
7: Department of Mechanical Engineering, Ehime University, 2-5 Bunkyo-cho, Matsuyama 790-8577, Japan
8: National Research Nuclear University (MEPhI), Kashirskoe shosse 31, Moscow, 115409, Russian Fedration
9: Fuji Die Co., Ltd., 2-17-10 Shimomaruko, Ohta-ku, Tokyo 146-0092, Japan
10: Earth-Life Science Institute, Tokyo Institute of Technology, 2-12-1-1E-1 Ookayama, Meguroku, Tokyo 152-8500, Japan

*, Corresponding author, Norimasa Nishiyama, email: norimasa.nishiyama@desy.de

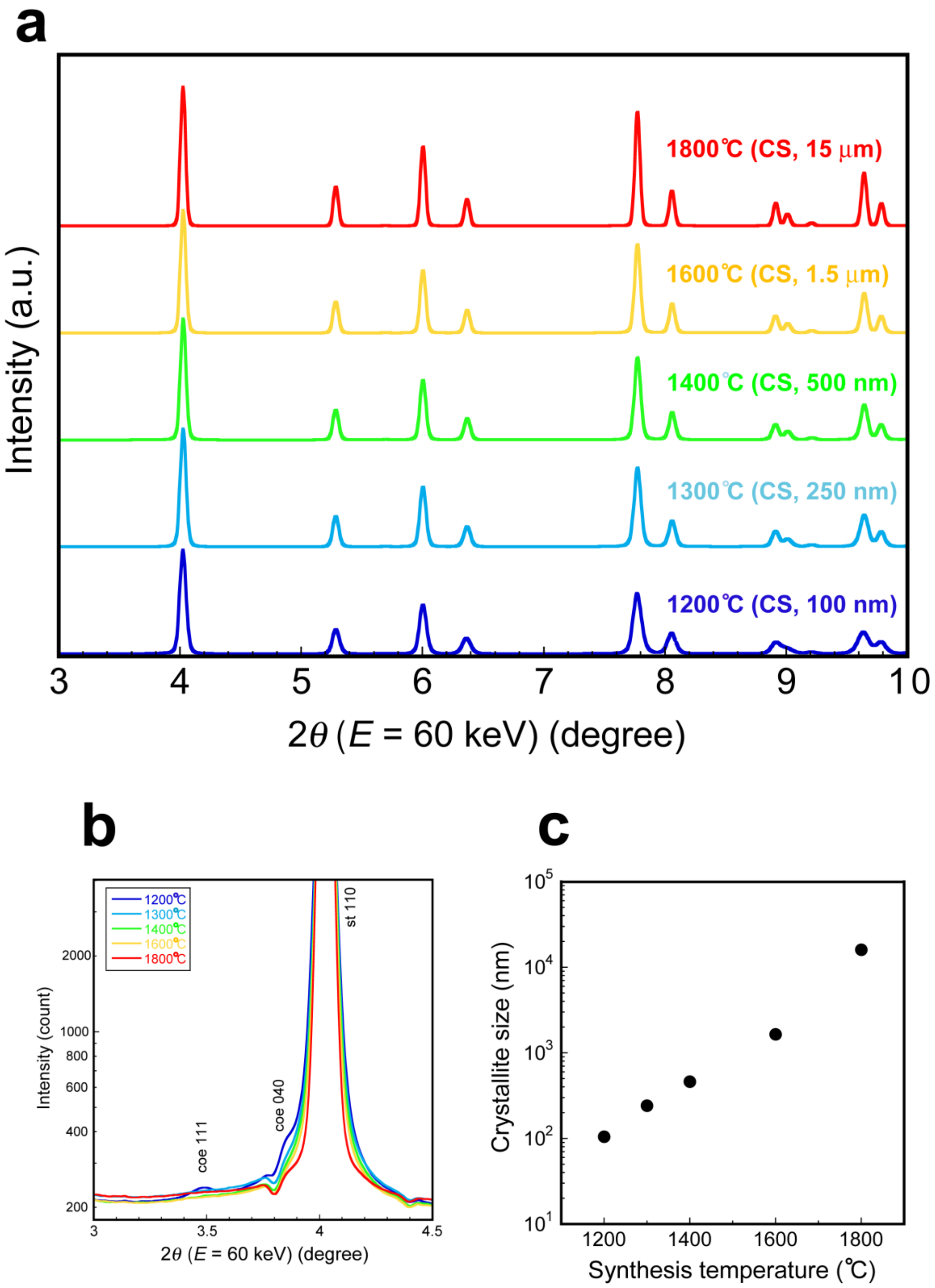

Figure S1 | Results of X-ray diffraction measurements obtained at P02.1 at PETRA III, Germany. a, Representative X-ray diffraction patterns of stishovite polycrystals synthesized at different temperatures at 15 GPa. All the peaks shown in this figure are explained by stishovite. Note that peak width decreases with synthesis temperature. **b,** Data to show presence of a small amount of coesite (0.2 vol%) in a sample synthesized at 1200°C. Samples synthesized above 1300°C are pure stishovite polycrystals. **c**, Refined crystallite size as a function of synthesis temperature.

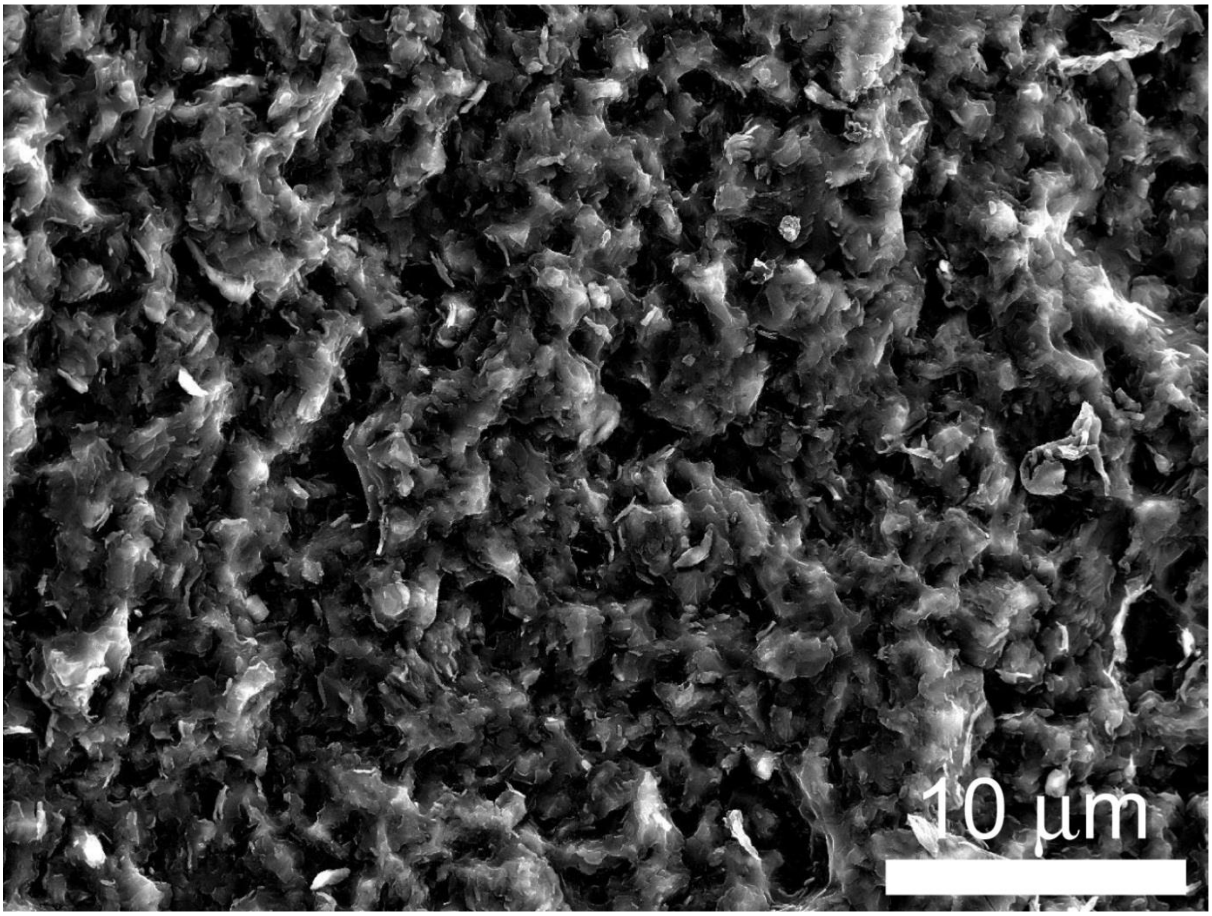

Figure S2 | Microstructure of fracture surface of a stishovite polycrystals synthesized at 1300°C. Worm-like textures are not local features. The whole fracture surface is completely filled with this texture.

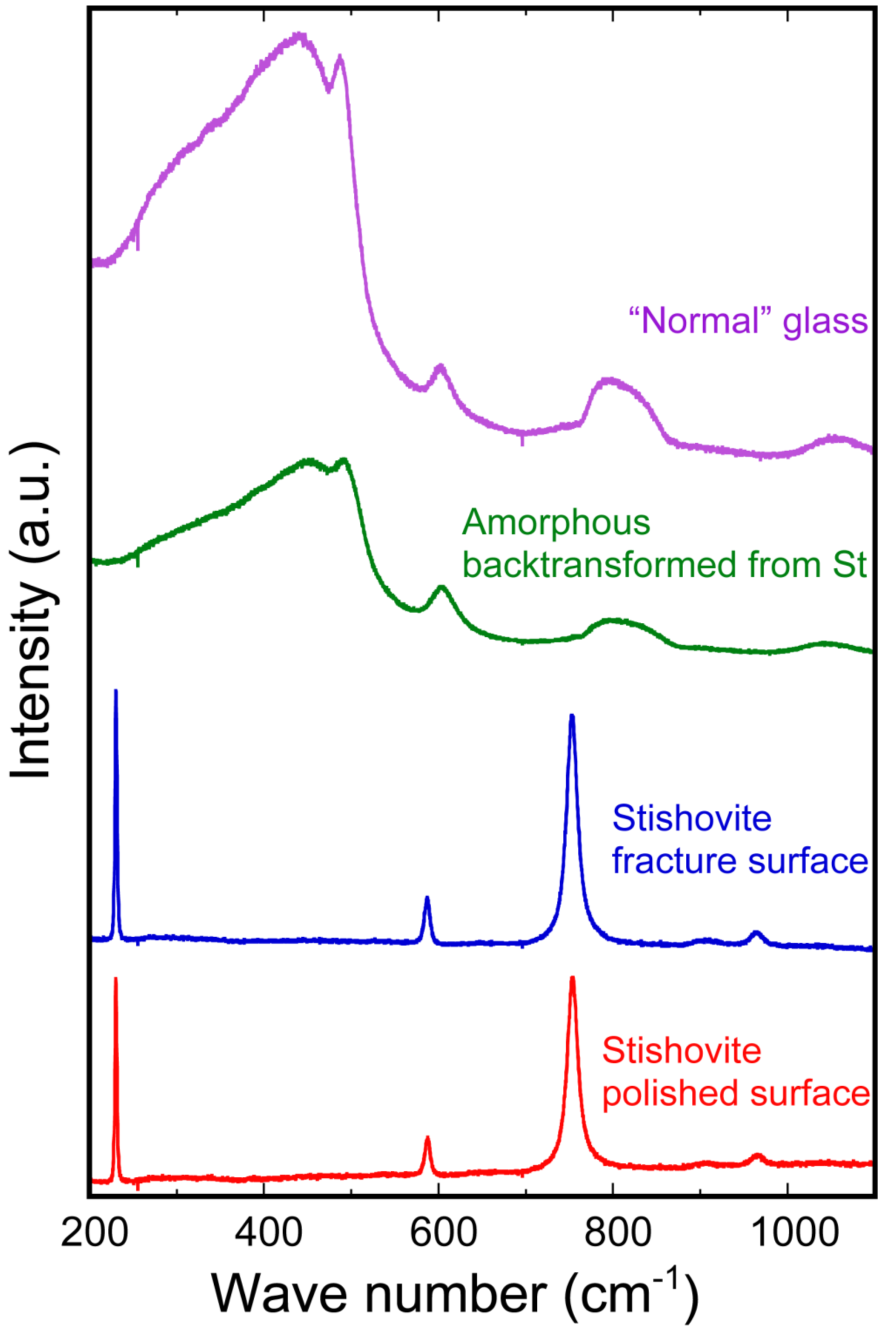

Figure S3 | Raman spectra obtained from polished and fracture surfaces of stishovite polycrystals. Stishovite polished surface, a polished surface of a stishovite poly-crystal synthesized at 1700°C. Stishovite fracture surface, a fracture surface of a stishovite poly-crystal synthesized at 1300°C. Amorphous backtransformed from St, a stishovite poly-crystal synthesized at 1600°C was heated up to 1000°C in air and the stishovite was transformed to an amorphous phase. Thin amorphous layers that are observed by TEY-XANES measurements on the polished and fracture surfaces cannot be detected by conventional Raman spectroscopy. The excitation laser is a Nd:YAG laser (wave length = 532 nm). All the spectra were obtained at Raman Spectroscopy Lab, GFZ Potsdam, Germany.

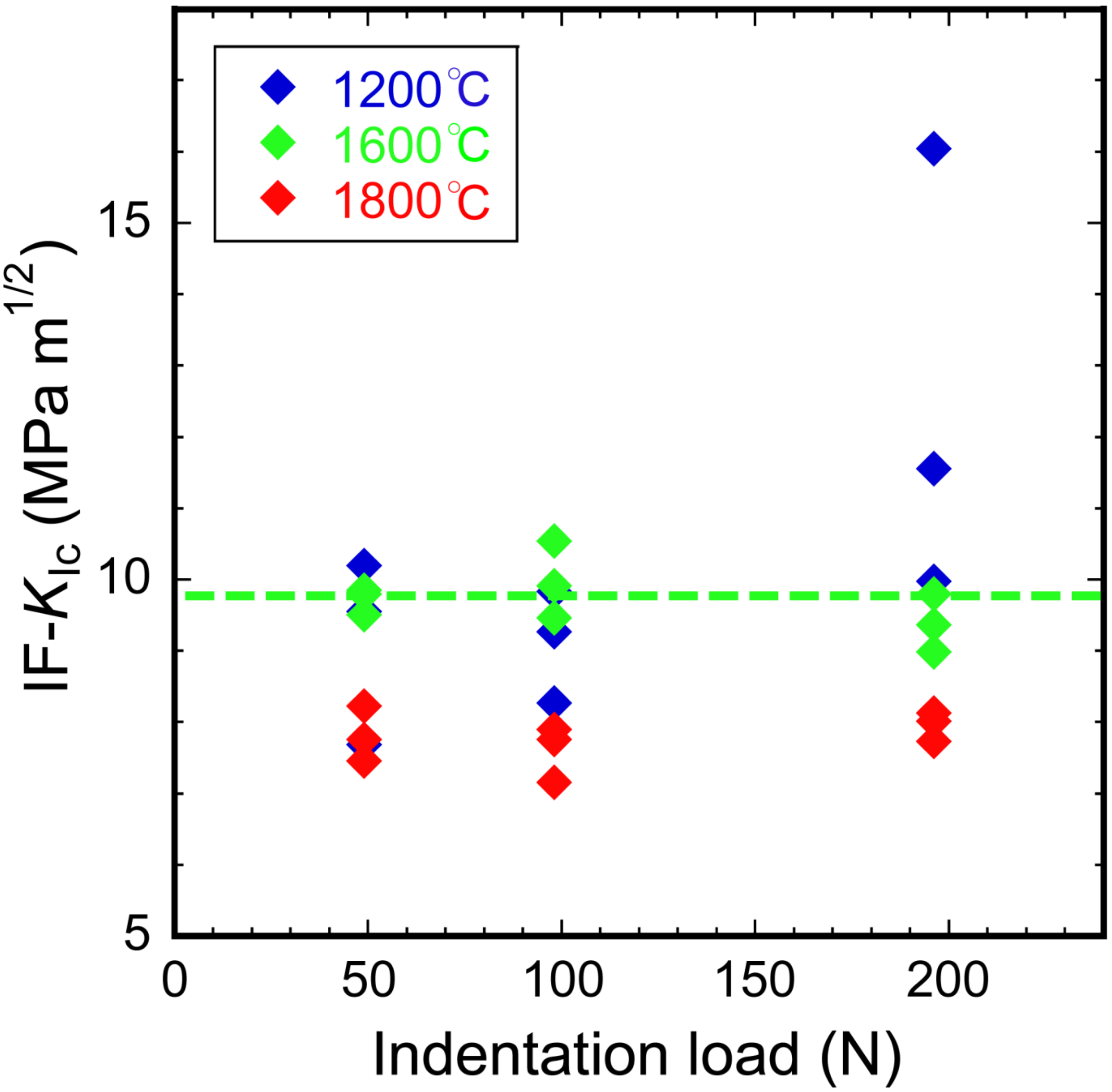

Figure S4 | Indentation fracture toughness (IF-K_{Ic}) of stishovite polycrystals as a function of indentation load. The measurements were performed at 49, 98, and 196 N. The IF-K_{Ic} values of a stishovite polycrystal (SP) synthesized at 1200°C are scattered. The average value at 196 N is 13 MPa m$^{1/2}$ whereas those at 49 and 96 N are less than 10 MPa m$^{1/2}$. The averaged IF-K_{Ic} of all the measurements for a SP synthesized at 1200°C is 10.3 ± 2.4 MPa m$^{1/2}$. The scatter of IF-K_{Ic} data obtained from a SP synthesized at 1200°C might be attributed to the presence of a small amount of coesite (0.2 vol%) (Fig. S1b). IF-K_{Ic} values of SPs synthesized above 1300°C (pure stishovite polycrystals) are almost constant with indentation load.

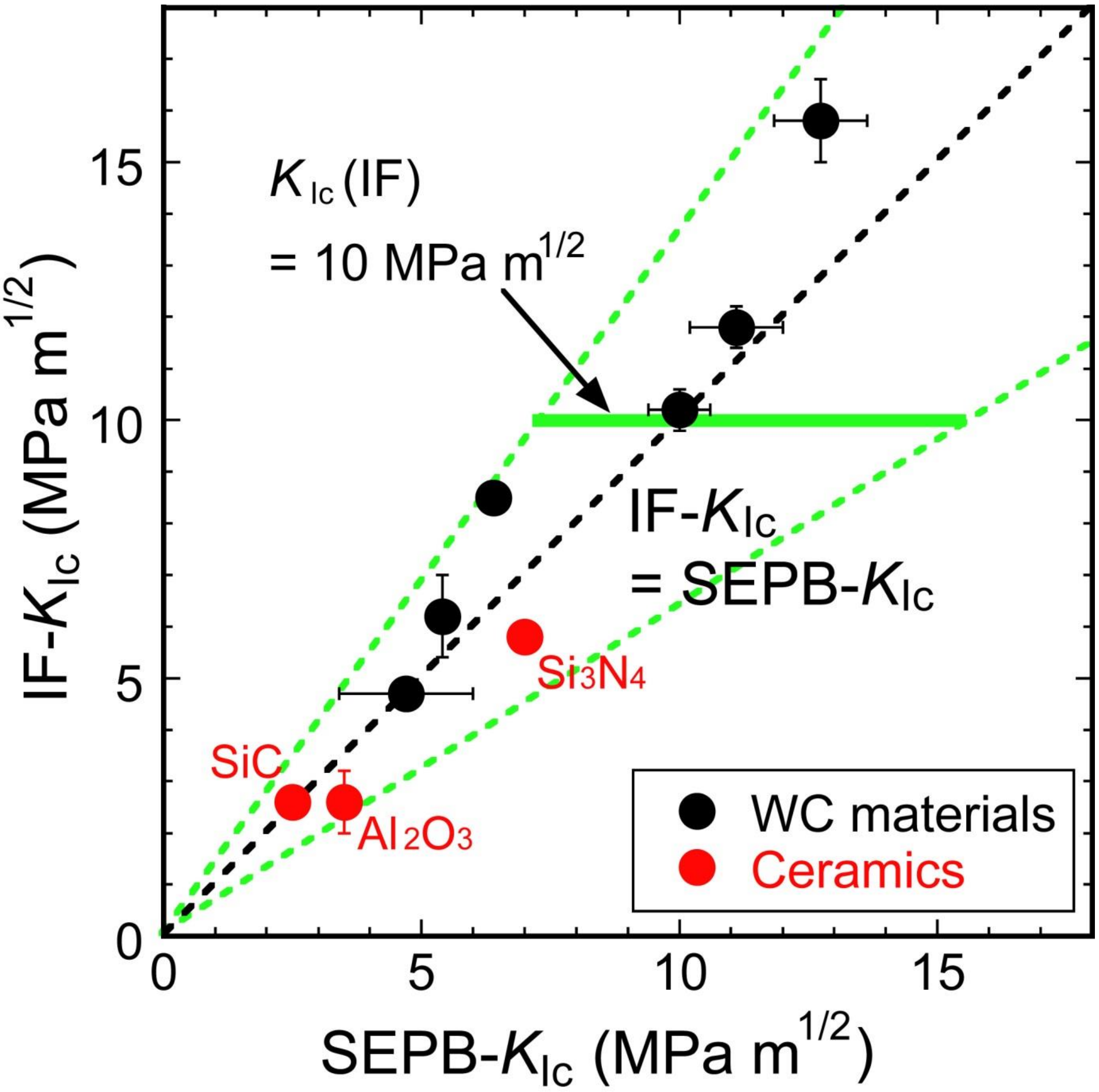

Figure S5 | Relationship between fracture toughness determined by an indentation fracture method (IF-K_{Ic}) and that by a single edge precracked beam (SEPB) method. The SEPB method is one of the most accurate methods to measure fracture toughness of monolithic ceramics. The black dashed-line represents the relation of IF-K_{Ic} = SEPB-K_{Ic}. Ceramics: Al_2O_3, alumina (A-479, Kyocera); SiC, silicon carbide (SC1000, Kyocera); Si_3N_4, silicon nitride (SN-240, Kyocera). WC materials from low to high fracture toughness: TJS-02 (Fujilloy); TF05 (Fujilloy); F08 (Fujilloy); D10 (Fujilloy); VD15 (Fujilloy); D40 (Fujilloy). IF-K_{Ic} = 10 MPa $m^{1/2}$ corresponds to SEPB-K_{Ic} = 7-15 MPa $m^{1/2}$ (the green solid line).

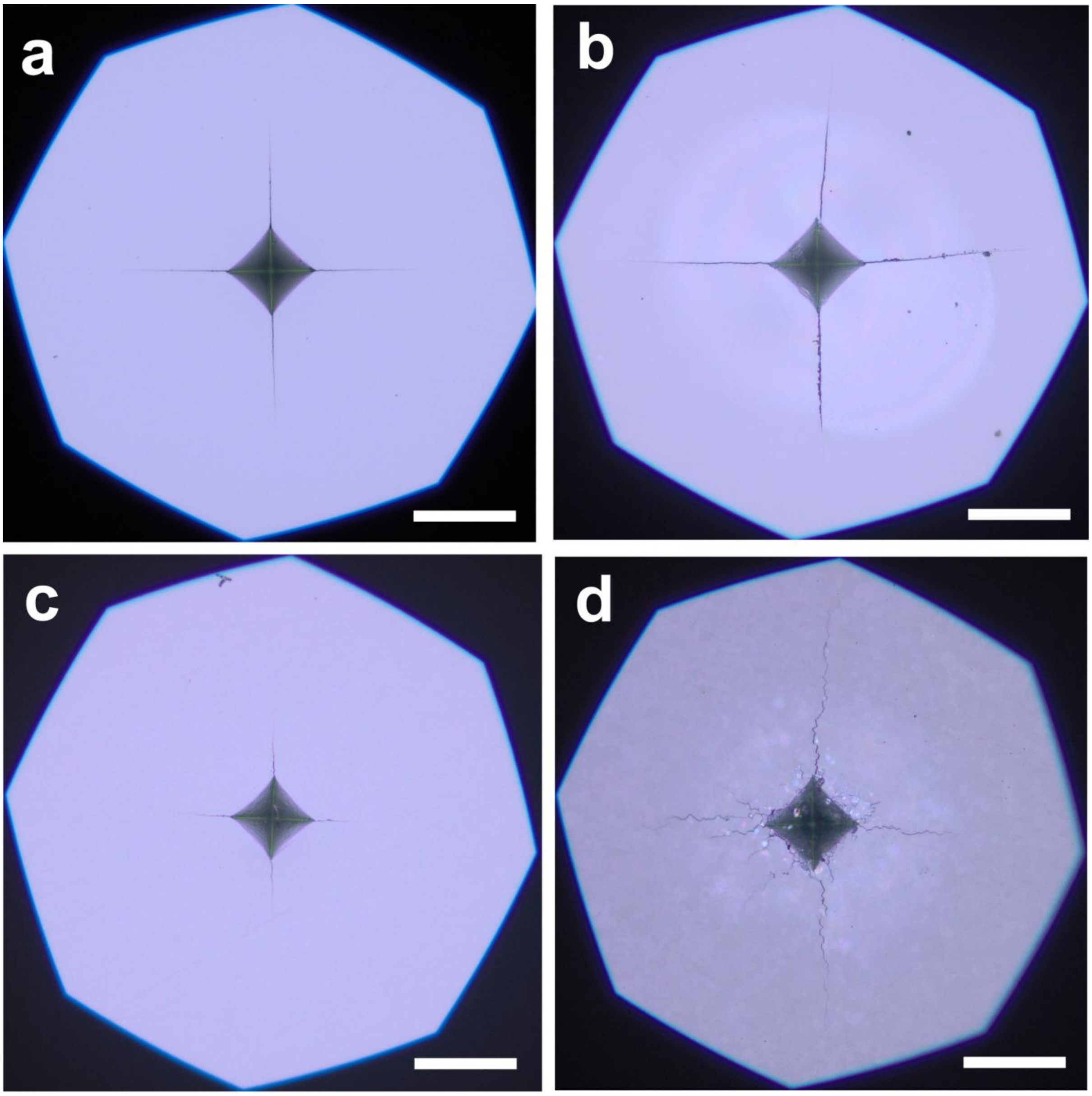

Figure S6 | Optical micrographs of Vickers indentation traces of a WC material (a), a transparent polycrystalline alumina (b), and stisohovite polycrystals (c, d). **a**, a WC material, TF05 (Fujilloy, Vickers hardness (H_V) = 22.4 GPa, IF-K_{1c} = 6.2 MPa m$^{1/2}$). **b**, a transparent polycrystalline alumina synthesized using a spark plasma sintering equipment [S1, S2]: H_V = 20.6 ± 0.3 GPa and 2.3 ± 0.1 MPa m$^{1/2}$. **c**, a stishovite polycrystal synthesized at 1300°C. **d**, a stishovite polycrystal synthesized at 1900°C (**c** and **d** are the same as Fig. 5c and Fig. 5d in the main text. These are shown for comparison). All the indentation traces were obtained at an applied load of 98 N and the scale bars represent 100 μm.

Details of XANES simulation

The XANES simulations were carried out by first-principles method with a core-hole effect included. [S3] The calculated models were based on 72-atom 2×2×3 and 2×2×2 supercell for stishovite and α-quartz, respectively. The atomistic structure of models were calculated with the projector augmented wave (PAW) method [S4] implemented in Vienna *ab-initio* simulation package (VASP) code.[S5, S6] The cut-off energy of the plane waves was set to 600 eV. The *k*-point sampling was carried out by Γ-centered 2×2×2 Monkhorst-Pack mesh. [S7] The exchange-correlational functional was given by the generalized gradient approximation proposed by Perdew, Burke and Ernzerhof (GGA-PBE). [S8] The structural parameters were fully relaxed until the residual forces and stress became less than 0.03 eV/Å and 0.3 GPa. In this condition, the lattice constants were overestimated by about 2%, but this was usual GGA tendency. The convergence tests were carried out by comparing more severe conditions, 800 eV of cut-off energy and 4×4×4 *k*-mesh and the total energy was converged within 5 meV atoms.

Theoretical Si-*K* XANES spectra were calculated by augmented plane-wave + local orbital (APW+*lo*) method implemented in WIEN2k code [S9] with a core-hole effect included. The cut-off parameter, $R_{MT}\cdot K_{MAX}$, were set to 6.0 bohr·Ry$^{1/2}$. The R_{MT}s were set to 1.30 and 1.60 bohr for Si and O. The models, *k*-mesh and exchange-correlational functional were the same conditions as those in the structural calculations. The calculated XANES spectra were broadened by Lorentz function with the natural width of Si-*K* shell. [S10] The transition energy was corrected via the alignment of the strongest peak of each substance. We also performed convergence tests by comparing more severe conditions, 7.0 bohr· Ry$^{1/2}$ and 4×4×4 *k*-mesh. It was confirmed that spectra were quantitatively well converged in the present conditions.

[S1] N. Nishiyama et al., *Scripta Mater.* **69**, 362 (2013)

[S2] B. Kim, K. Hiraga, K. Morita, H. Yoshida, *Scripta Mater.* **57**, 607 (2007)

[S3] I. Tanaka, T. Mizoguchi, and T. Yamamoto, *J. Am. Ceram. Soc.* **88**, 2013-2029 (2005).

[S4] P. E. Blöchl, *Phys. Rev. B* **50**, 17953 (1994).

[S5] G. Kresse and J. Furthmüller, *Phys. Rev. B* **54**, 11169 (1996).

[S6] G. Kresse and D. Joubert, *Phys. Rev. B* **59**, 1758 (1999).

[S7] H. J. Monkhorst and J. D. Pack, *Phys. Rev. B* **13**, 5188 (1976).

[S8] J. P. Perdew, K. Burke, and M. Ernzerhof, *Phys. Rev. Lett.* **77**, 3865 (1996).

[S9] P. Blaha, K. Schwarz, G. Madsen, D. Kvasicka, and J. Luitz, WIEN2k, An Augmented Plane Wave Local Orbitals Program for Calculating Crystal Properties, ISBN 3-9501031-1-2. Karlheinz Schwarz, Techn. Universitat Wien, Austria, 2001.

[S10] M. O. Krause and J. H. Oliver, *J. Phys. Chem*. Ref. Data 8, 329 (1979).
